# A Giant Proliferating Trichilemmal Tumor of the Perineum with Pelvic Extension: Transperineal Resection Facilitated by Cyst Fluid Redistribution and Enhanced Perineal Expansion

**DOI:** 10.70352/scrj.cr.26-0656

**Published:** 2026-10-24

**Authors:** Masatsugu Kojima, Toru Miyake, Soichiro Tani, Keiji Muramoto, Takeshi Sonoda, Kenji Isogawa, Sachiko Kaida, Katsushi Takebayashi, Hiromitsu Maehira, Haruki Mori, Reiko Otake, Nobuhito Nitta, Tomoharu Shimizu

**Affiliations:** 1 Department of Gastrointestinal and General Surgery, Shiga University of Medical Science, Otsu, Shiga, Japan; 2 Department of Pathology, Shiga University of Medical Science, Otsu, Shiga, Japan; 3 Medical Safety Section, Shiga University of Medical Science Hospital, Otsu, Shiga, Japan

**Keywords:** proliferating trichilemmal tumor, perineum, pelvic extension, giant cystic tumor, surgical resection, transperineal approach, transperineal resection

## Abstract

**INTRODUCTION:**

Proliferating trichilemmal tumor (PTT) is a rare neoplasm arising from the outer root sheath of the hair follicle and typically occurs on the scalp of elderly women. Perineal occurrence is extremely rare, and, to our knowledge, no giant perineal PTT extending into the retroperitoneal pelvic space has been previously reported.

**CASE PRESENTATION:**

A 77-year-old man presented with a giant perineal mass that had gradually enlarged over 10 years and rapidly increased in size during the preceding 2 years. CT and MRI demonstrated a 35 cm dumbbell-shaped cystic lesion extending from the perineum into the retroperitoneal pelvic space, with papillary projections arising from the cyst wall. Malignant transformation could not be ruled out preoperatively. Tumor resection was achieved through the transperineal approach, facilitated by gravitational redistribution of the cystic contents and expansion of the perineal component, followed by laparoscopic inspection confirming no intra-abdominal or pelvic injury. Histopathological examination demonstrated trichilemmal keratinization without cytological atypia or evidence of malignancy, leading to a diagnosis of benign PTT. The postoperative course was uneventful.

**CONCLUSION:**

This case represents one of the largest PTTs reported to date and highlights the exceptional rarity of a perineal PTT with pelvic extension. A transperineal approach may provide an effective surgical option for selected noninvasive cystic lesions extending into the retroperitoneum.

## Abbreviations

α-SMA
: α-smooth muscle actin
ADC
: apparent diffusion coefficient
CEA
: carcinoembryonic antigen
DWI
: diffusion-weighted imaging
PTT
: proliferating trichilemmal tumor
SCC
: squamous cell carcinoma antigen

## INTRODUCTION

PTT is a rare adnexal neoplasm arising from the outer root sheath of the hair follicle and was first described by Wilson-Jones in 1966.^1,2)^ It predominantly occurs on the scalp of elderly women and is characterized histopathologically by trichilemmal keratinization. Although most PTTs are benign, malignant transformation has occasionally been reported, making complete surgical excision the treatment of choice. PTTs arising outside the scalp are uncommon, and involvement of the perineum is exceptionally rare. Moreover, to our knowledge, no giant perineal PTT extending into the retroperitoneal pelvic space has been reported. Such an unusual location and extensive tumor growth pose considerable diagnostic and surgical challenges.

We report an exceptionally rare case of a giant perineal PTT with retroperitoneal extension that was successfully resected through a transperineal approach. In addition, we describe an effective surgical technique that took advantage of the marked perineal expansion of the cyst and gravitational redistribution of the cystic contents to facilitate caudal descent of the pelvic component and enable resection from the perineal side.

## CASE PRESENTATION

A 77-year-old man presented with a perineal mass. He had initially noticed the lesion 10 years earlier but had not sought medical evaluation. The mass gradually enlarged over time, with rapid growth beginning 2 years prior to presentation, and eventually became large enough to interfere with sitting. He sought medical attention after developing a skin erosion over the lesion due to repeated friction. His medical history was notable only for hypertension.

On physical examination, a giant mass was observed in the left buttock and perineal region (**Fig. 1A**). The tumor was relatively soft and elastic on palpation. In the prone position, the mass occupied the left gluteal region and extended toward the perineum. In the Sims’ position, the tumor did not obstruct the anus; however, digital rectal examination revealed extramural compression extending from the left side to the posterior wall of the rectum without evidence of luminal stenosis (**Fig. 1B**). In the supine position, the mass caused marked protrusion of the lower abdomen because of compression from the perineal side (**Fig. 1C**). Laboratory data showed mildly elevated tumor markers, with CEA at 6.8 ng/mL and SCC at 10.5 ng/mL. Other laboratory findings were within normal limits, with no evidence of inflammation, malnutrition, or anemia. CT revealed a giant cystic mass extending from the pelvic cavity to the subcutaneous tissue of the perineum (**Fig. 2**). The lesion measured 35 cm in the craniocaudal dimension and exhibited a dumbbell-shaped configuration. The margins were well defined, with a slightly thickened capsule. Septations were observed within the gluteal portion of the mass. The tumor displaced the intra-abdominal organs cranially, the rectum to the right side, and the male genitalia ventrally. Despite its massive size, no apparent invasion of the intra-abdominal organs, rectum, or gluteal muscles was identified. MRI demonstrated a giant cystic lesion showing high signal intensity on T2-weighted images and low signal intensity on T1-weighted images (**Fig. 3**). The margins were well defined, and the lesion was surrounded by a thick fibrous capsule. In addition, papillary projections arising from the cyst wall and protruding into the lumen showed low signal intensity on T2-weighted images and mildly increased signal intensity on DWI, with slightly decreased ADC values. These components showed slight diffusion restriction, suggesting the presence of proliferative solid elements within the cystic lesion. On coronal T2-weighted imaging, the levator ani muscle was markedly stretched and displaced by the giant cyst (**Fig. 3D**). Because of the severe compression and distortion caused by the tumor, the precise spatial relationship among the individual pelvic floor structures could not be clearly delineated.

**Fig. 1:**
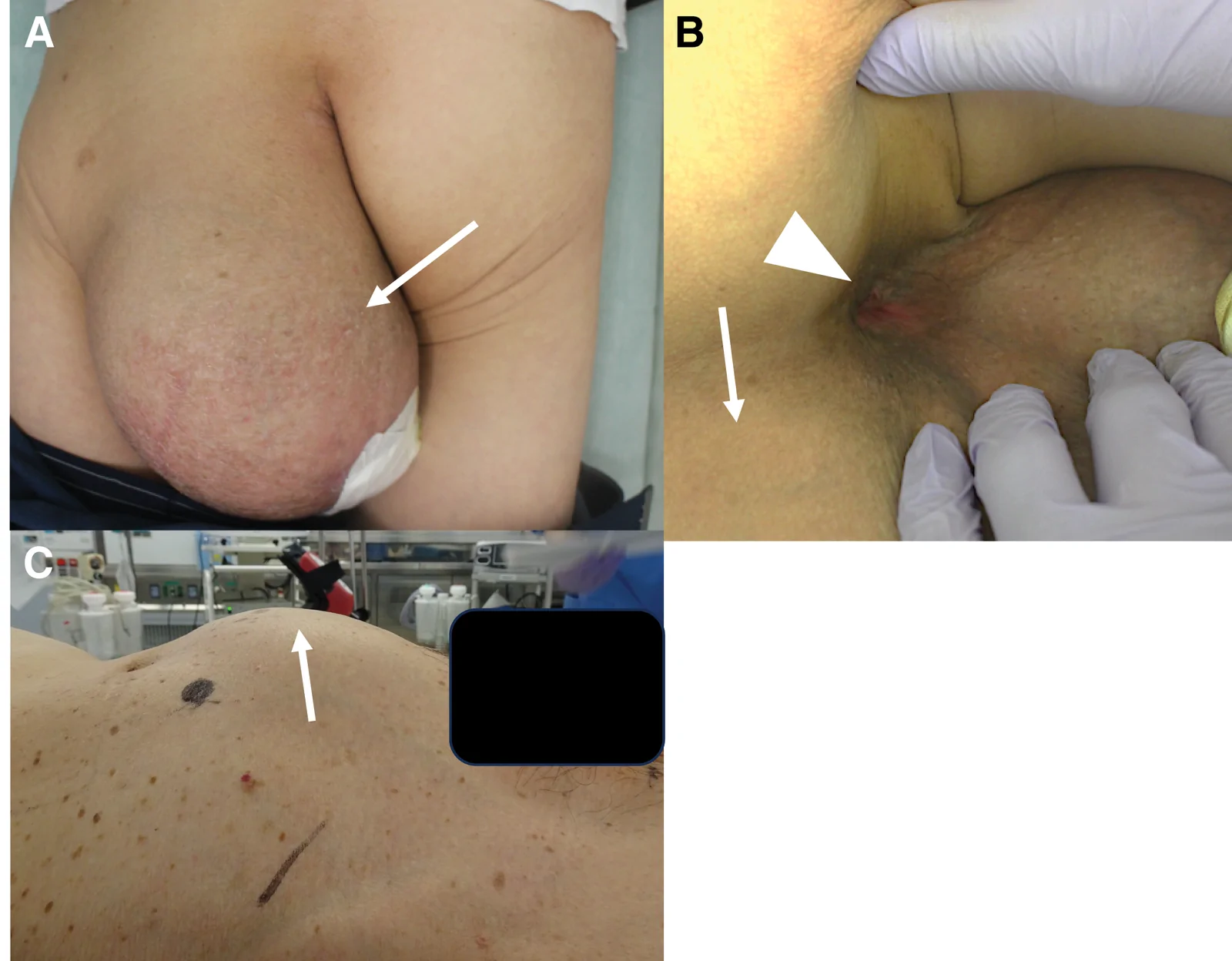
Clinical appearance of the giant perineal tumor. (**A**) Prone position. A relatively soft and elastic mass was observed on the left side of the buttock (arrow). (**B**) Sims’ position. The mass (arrow) did not obstruct the anus (arrowhead). Digital rectal examination revealed no stenosis; however, extramural compression was palpated from the left side to the posterior rectal wall. (**C**) Supine position. The mass (arrow) compressed and protruded into the lower abdomen.

**Fig. 2:**
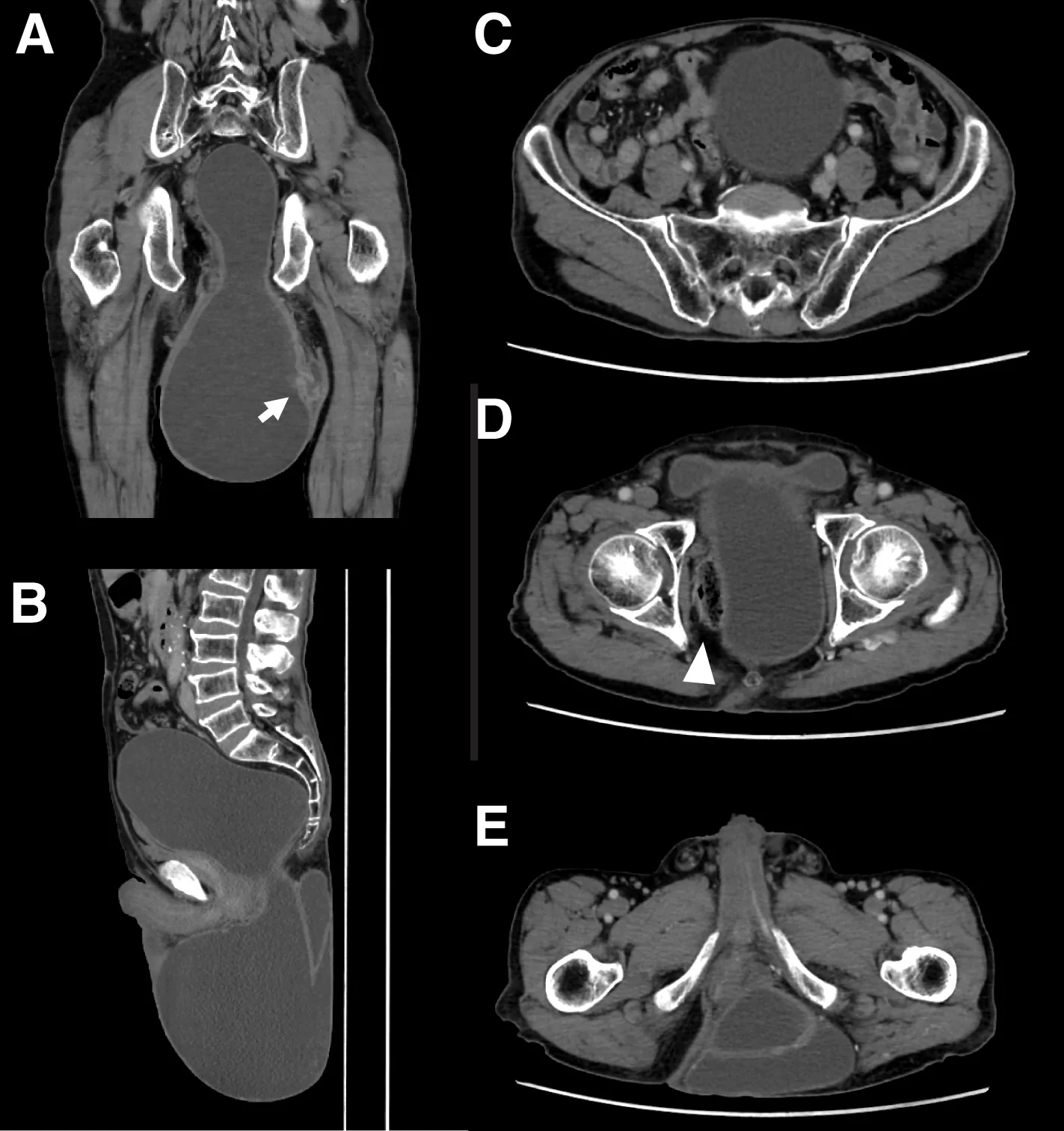
Equilibrium-phase contrast-enhanced CT findings. (**A**) Coronal view. CT revealed a giant cystic mass extending from the pelvic cavity to the subcutaneous tissue of the perineum. The lesion measured 35 cm in the craniocaudal dimension and exhibited a dumbbell-shaped configuration. The margins were relatively well defined, with a slightly thickened capsule. Septations were observed within the gluteal portion of the mass (arrow). (**B**) Sagittal view. Within the abdominal cavity, the mass displaced the intra-abdominal organs cranially. On the perineal side, the male genitalia were displaced ventrally by the tumor. The cyst exhibited a constriction at the level of the bony pelvic outlet. (**C**–**E**) Axial views from cranial to caudal levels. (**C**) No evidence of adhesion to or invasion of the intra-abdominal organs was identified. (**D**) The rectum was displaced to the right side by the cystic mass (arrowhead). The boundary between the rectum and the tumor was clearly preserved, with no evidence of invasive growth. (**E**) The cystic mass displaced the scrotum ventrally. No invasion into the gluteal muscles was observed.

**Fig. 3:**
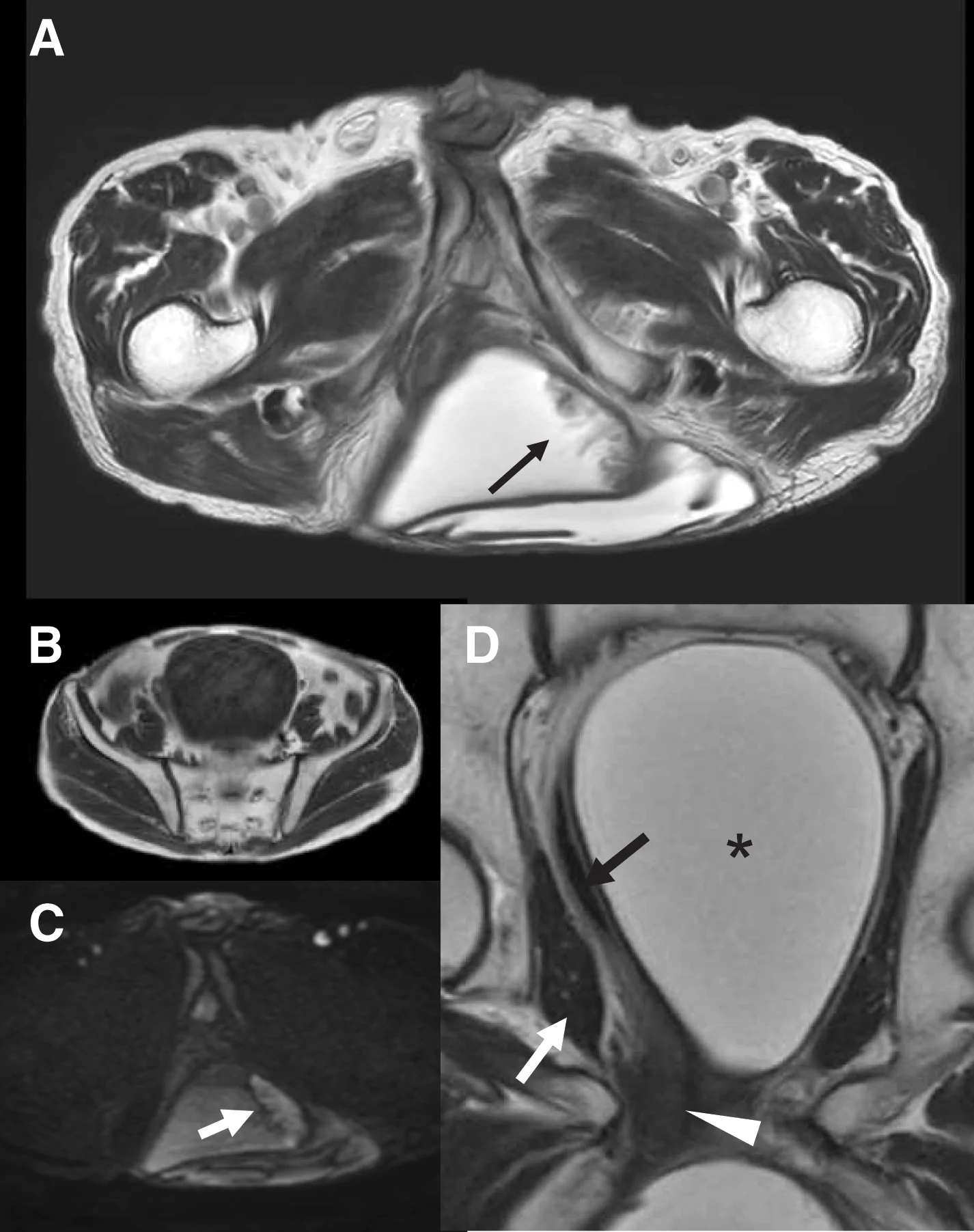
MRI findings. (**A**) MRI demonstrated a cystic lesion showing high signal intensity on T2-weighted images. The margins were well defined, and the lesion was surrounded by a thick fibrous capsule. Papillary proliferative components projecting into the lumen (arrow) exhibited low signal intensity. (**B**) The cystic lesion showed low signal intensity on T1-weighted images. (**C**) The papillary proliferative components projecting into the lumen (arrow) exhibited mildly increased signal intensity on DWI. (**D**) Coronal T2-weighted image demonstrating marked stretching and displacement of the levator ani muscle by the giant cyst. Because of severe compression and distortion, the precise spatial relationship among the individual pelvic floor structures was difficult to delineate. The black arrow indicates the levator ani muscle, the white arrow indicates the obturator internus muscle, the arrowhead indicates the anal canal, and the asterisk indicates the cyst. DWI, diffusion-weighted imaging

The primary origin of the lesion—whether from the pelvic cavity or the perineum—was unclear. The differential diagnoses included dermoid cyst, epidermoid cyst, duplication cyst, and tailgut cyst. A biopsy was not performed due to the risk of cyst rupture and spillage of cystic contents, as well as the technical difficulty of targeting the papillary components located deep within the pelvis. The papillary protruding components raised concern for possible malignant transformation. In addition, as the mass had begun to interfere with ADL, surgical en bloc resection was planned.

Our initial operative plan was to begin with transperineal dissection and subsequently proceed to laparoscopic dissection from the abdominal side. By creating the perineal dissection space first, we anticipated that placement of gauze within this space would help maintain the distal dissection cavity and facilitate connection of the abdominal and perineal dissection planes.

Under general anesthesia, the patient was placed in the jackknife position (**Fig. 4**). A circumferential skin incision was made at a relatively high level on the protruding mass to preserve sufficient perineal skin for subsequent wound closure. After circumferential exposure of the cyst wall, dissection was continued cranially along the cyst wall, separating it from the surrounding tissues. In the perianal region, this dissection proceeded within the ischiorectal fossa, with particular care taken to avoid injury to the external anal sphincter. No evidence of invasion into the surrounding tissues was observed. As the cyst was freed from the surrounding compressed tissues, the extracorporeal portion was repositioned into a dependent position between the legs and lowered below the level of the pelvic component, without tilting the operating table and with the patient remaining in the jackknife position. This allowed the cystic contents to shift toward the extracorporeal/perineal component under gravity, causing the pelvic component to progressively collapse and descend into the perineal operative field, where further dissection was performed under direct vision (**Fig. 5**). Throughout the procedure, the cyst wall remained intact, and no intraoperative rupture or spillage of the cystic contents occurred. The location of the papillary protruding component could be identified by direct palpation of the cyst wall. In this area, the dissection line was intentionally placed outside the cyst wall so that a rim of surrounding soft tissue was included with the specimen rather than dissecting directly along the cyst wall. Ultimately, the cyst was dissected from the surrounding fascia on the cranial side, which was presumed to include the endopelvic fascia and retroperitoneal tissue. The patient was then placed in the supine position, and the planned laparoscopy was performed for intra-abdominal and pelvic inspection. This was undertaken to confirm the absence of injury to the rectum, peritoneum, or other pelvic organs, as well as to exclude additional intra-abdominal or pelvic lesions potentially associated with the cystic lesion in other organs and residual cystic or daughter lesions. After the small intestine was displaced from the pelvis, no injury to the rectum or other pelvic organs was observed (**Fig. 6A**). No additional intra-abdominal or pelvic lesions, whether related or unrelated to the cystic lesion, were identified. Elevation of the peritoneum revealed marked stretching of the retroperitoneum resulting from compression by the tumor (**Fig. 6B**). The circular defect was closed in a T-shaped fashion after excision of the dogears. The total operative time was 407 min, and the estimated blood loss was 212 mL. The transperineal procedure from the beginning of perineal dissection to tumor removal required 186 min, perineal wound reconstruction by a plastic surgeon required 88 min, and subsequent laparoscopic inspection required 21 min. Additional time was required for intraoperative repositioning from the supine to the jackknife position and subsequently back to the supine position, as well as for the associated preparation and redraping.

**Fig. 4:**
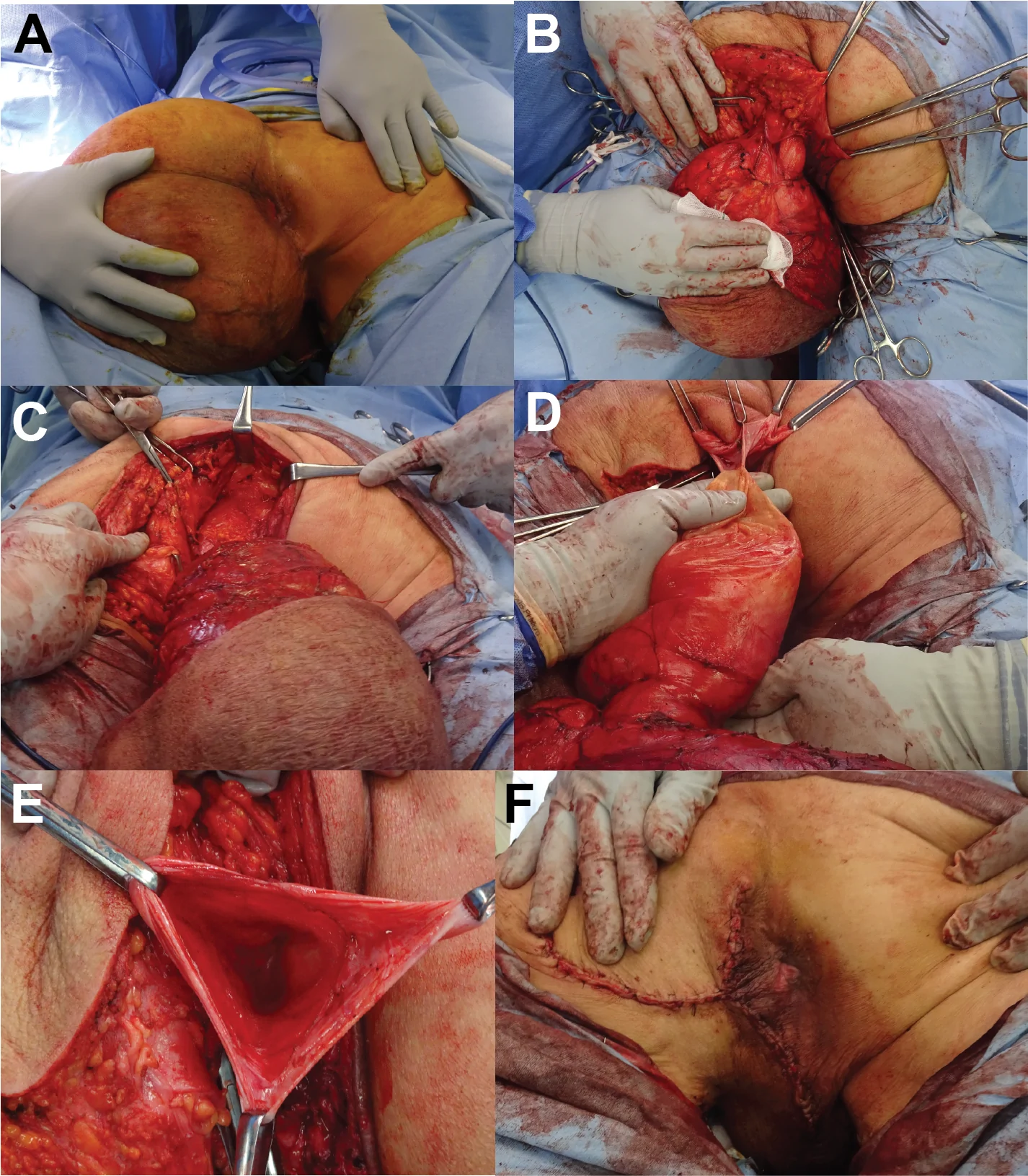
Intraoperative findings. (**A**) The patient was placed in the jackknife position. The mass was located on the left buttock, and lateral retraction of the mass to the left allowed visualization of the anus. (**B**) A circumferential skin incision was made at a relatively high level on the protruding mass to preserve sufficient perineal skin for subsequent wound closure. The cyst wall was circumferentially exposed and carefully dissected from the surrounding tissues. (**C**) Dissection was continued cranially along the cyst wall within the ischiorectal fossa, with particular care taken to avoid injury to the external anal sphincter and the rectum. No evidence of tumor invasion into the surrounding tissues was observed. (**D**) With the patient remaining in the jackknife position and without tilting the operating table, the extracorporeal portion of the cyst was repositioned into a dependent position between the legs and lowered below the level of the pelvic component. Gravity facilitated redistribution of the cystic contents toward the extracorporeal/perineal component, allowing the pelvic component to progressively collapse and descend into the operative field. (**E**) After tumor resection, the cyst had been separated from the surrounding fascia, which was presumed to include the endopelvic fascia and retroperitoneal tissue. (**F**) The circular defect was closed in a T-shaped fashion after excision of the dogears.

**Fig. 5:**
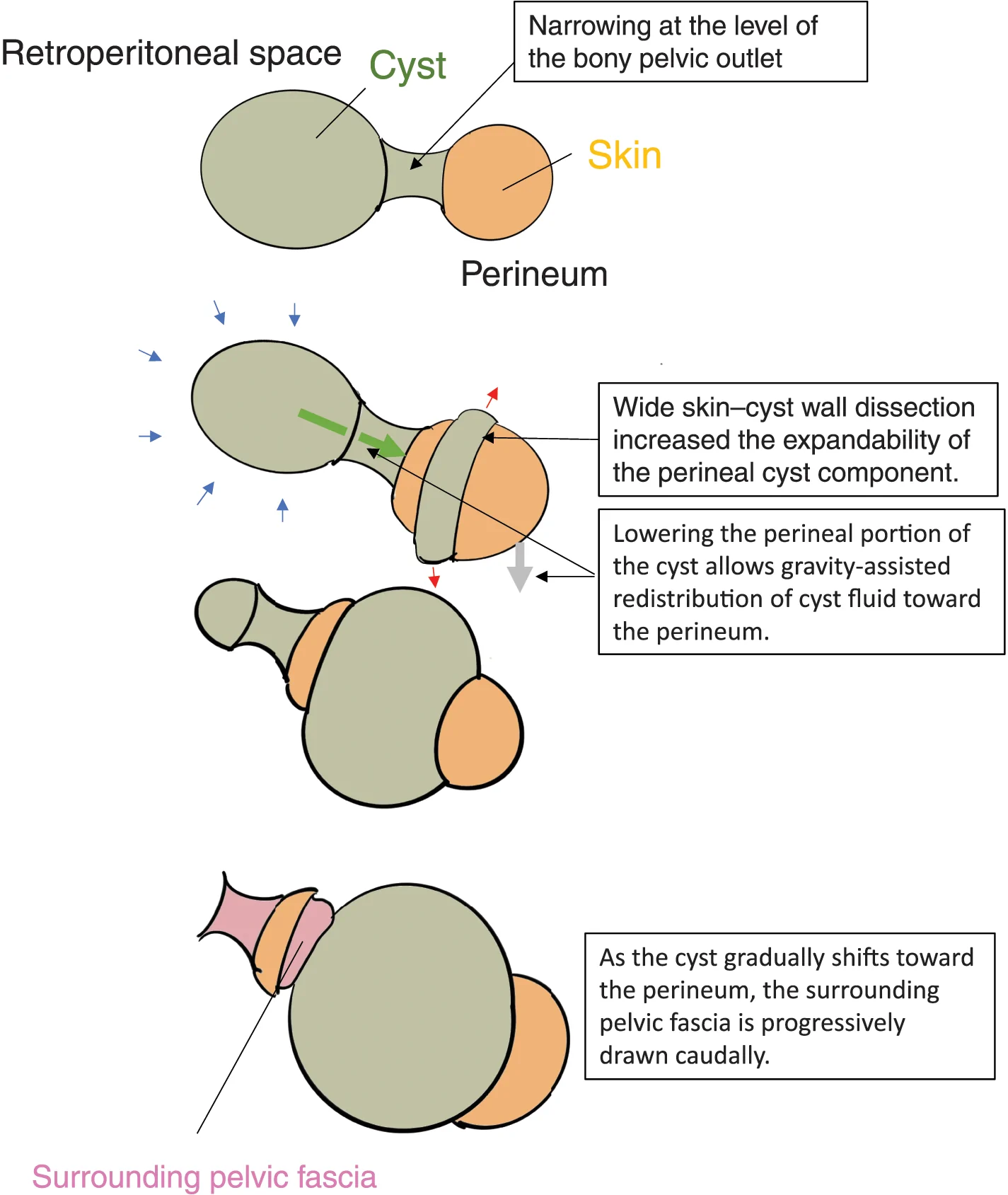
Schematic illustration of the surgical concept. Following circumferential skin incision and wide skin–cyst wall dissection, the perineal cyst component became more mobile and expandable. With the patient remaining in the jackknife position and without tilting the operating table, the extracorporeal/perineal component was repositioned into a dependent position between the legs and lowered below the level of the pelvic component. Gravity facilitated redistribution of the cystic contents toward the perineal component. As the pelvic component progressively collapsed, it descended into the perineal operative field, enabling safe dissection under direct vision and tumor resection through the transperineal approach.

**Fig. 6:**
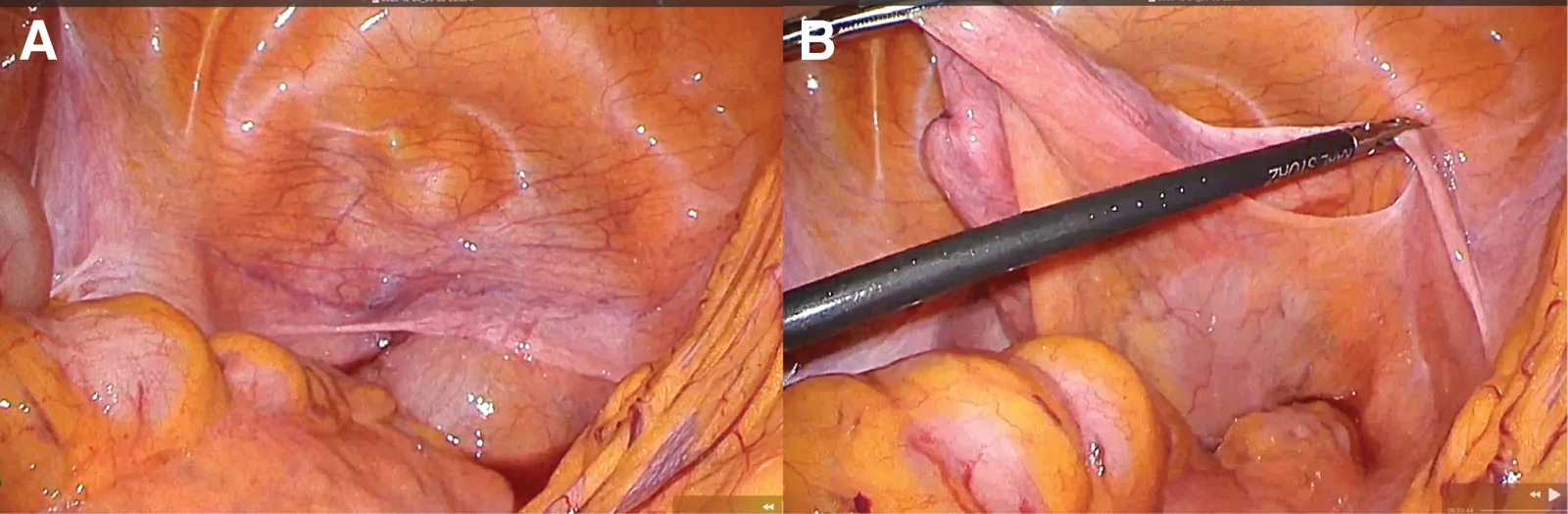
Laparoscopic inspection of the pelvic cavity after tumor resection. (**A**) After the small intestine was displaced from the pelvic cavity, laparoscopic inspection confirmed the absence of injury to the rectum or other pelvic organs and revealed no additional intra-abdominal or pelvic lesions. (**B**) Elevation of the peritoneum revealed marked stretching of the retroperitoneum resulting from compression by the tumor.

The postoperative course was uneventful, and the patient was discharged on POD 6. He remains free of recurrence at 6 months after surgery.

Gross examination of the resected specimen revealed a cystic lesion partially covered with overlying skin (**Fig. 7A**). The tumor was dumbbell-shaped and narrowed at the level of the bony pelvic outlet. Upon sectioning the tumor, the lesion consisted of a thick, flat cyst wall with a yellow to dark-yellow appearance and papillary projections protruding into the lumen (**Fig. 7B**). The cyst contained a viscous, whitish translucent fluid with yellow, friable debris (**Fig. 7C**). Histopathological examination demonstrated that the cyst wall was lined by squamous epithelium with hyperkeratosis (**Fig. 8A**). The squamous epithelium showed proliferation with trichilemmal keratinization, characterized by an abrupt transition from the stratum spinosum to keratin without an intervening stratum granulosum (**Fig. 8B**). In the papillary regions, epidermal papillary growth with marked hyperkeratosis was observed (**Fig. 8C**). Neither cytological nor nuclear atypia was identified, and there was no histopathological evidence of malignancy (**Fig. 8D**). Immunohistochemical staining for α-SMA did not reveal a smooth muscle layer, and no histopathological features suggestive of a tailgut cyst were identified. Based on these findings, the lesion was diagnosed as a PTT without evidence of malignancy.

**Fig. 7:**
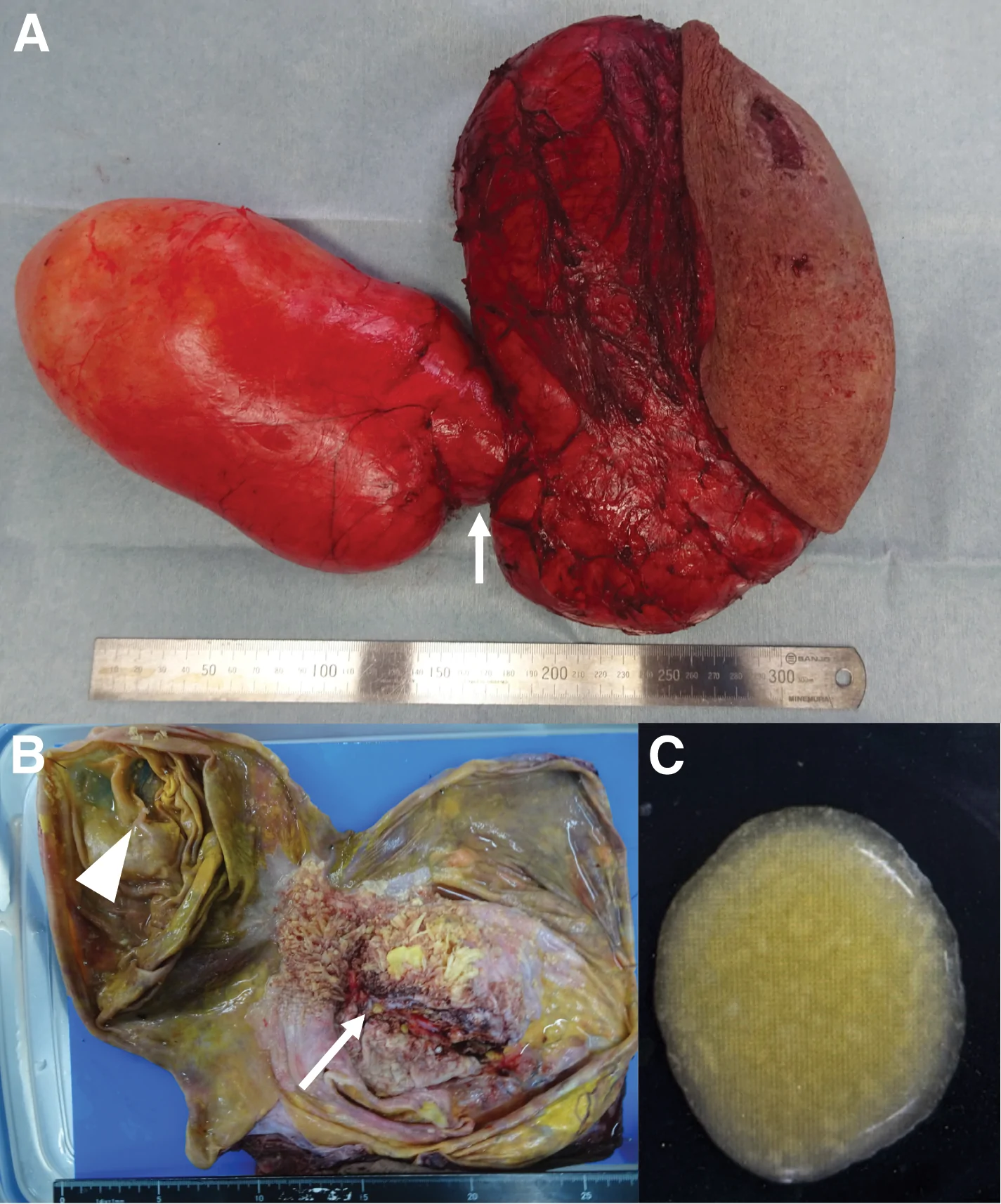
Gross findings. (**A**) The resected specimen, including the overlying skin. The cranial pelvic component was covered by a smooth cyst wall. The cyst was constricted at the level of the bony pelvic outlet (arrow). On the perineal side, the cyst was resected with a small amount of surrounding soft tissue attached. (**B**) The opened specimen. The cyst wall was thick and predominantly smooth on the inner surface. Focal internal septations were present (arrowhead). Yellowish papillary projections protruding into the cyst lumen were also observed (arrow). (**C**) Cystic contents. The cyst contained a viscous, whitish translucent fluid with yellow, friable debris.

**Fig. 8:**
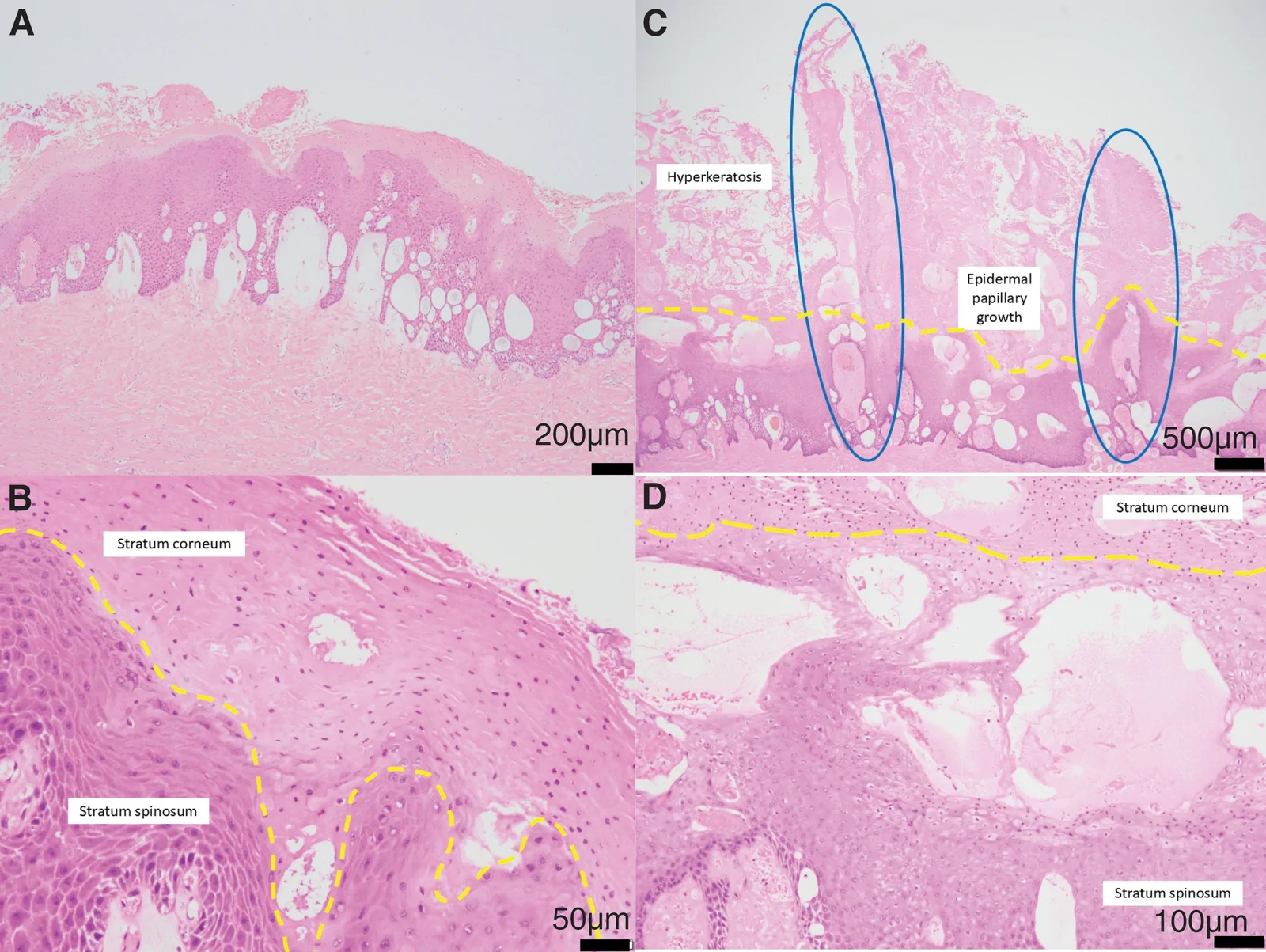
Histopathological findings. (**A**) Hematoxylin and eosin staining showed that the cyst wall was lined by squamous epithelium with hyperkeratosis (×40). (**B**) The squamous epithelium showed proliferation with trichilemmal keratinization, characterized by an abrupt transition from the stratum spinosum to the stratum corneum without an intervening stratum granulosum (×200). The dotted line indicates the transition from the stratum spinosum to the stratum corneum. (**C**) Epidermal papillary growth (blue circles) with marked hyperkeratosis (×20). (**D**) No cytological or nuclear atypia or histopathological evidence of malignancy was observed (×100).

## DISCUSSION

PTT is a rare adnexal neoplasm arising from the outer root sheath of the hair follicle.^1,2)^ Its histopathological hallmark is trichilemmal keratinization, characterized by an abrupt transition from nucleated epithelial cells to anucleate keratinized cells without an intervening granular layer, which is a key feature for diagnosis.^3)^ Although uncommon, malignant transformation can occur, with potential for local invasion as well as regional lymph node and distant metastases.^1)^ Clinically, rapid enlargement and intraluminal nodular or papillary components may raise suspicion of malignant transformation. In the present case, rapid tumor enlargement and the presence of papillary intraluminal components raised concern for malignant transformation. However, histopathological examination demonstrated neither infiltrative growth nor cytological atypia, confirming the diagnosis of benign PTT.

PTTs typically occur in elderly women, with more than 90% arising on the scalp. Occurrence in the perineum is exceedingly rare.^4)^ To date, only a limited number of PTTs arising in the perineum have been reported. The majority have occurred in the female vulva, whereas only 4 cases involving the perianal region or the ischiorectal fossa have been documented in the literature.^4–7)^ PTTs are generally small, with most measuring only a few centimeters in diameter. Giant PTTs are exceptionally uncommon. Among the published cases in which tumor size was clearly documented, only 3 measured more than 20 cm in diameter.^8–10)^ To the best of our knowledge, no previously reported PTT has exceeded 30 cm in diameter, making the present case one of the largest reported to date.

Histopathologically, malignant PTT is characterized by infiltrative growth, marked nuclear atypia, and geographic necrosis, with or without vascular or perineural invasion.^11)^ None of these features were observed in the present case. The differential diagnosis included tailgut cyst because of the lesion's location in the presacral–retroperitoneal space and its multilocular cystic morphology. Tailgut cysts are typically lined by various types of epithelia and often contain disorganized smooth muscle bundles within the cyst wall. In the present case, the characteristic trichilemmal keratinization, together with the absence of smooth muscle bundles as demonstrated by α-SMA immunohistochemistry, excluded this diagnosis.^12)^ Given the unusual perineal location, epidermoid cyst, dermoid cyst, and duplication cyst were also considered in the differential diagnosis. An epidermoid cyst was excluded by the absence of a granular layer and the presence of characteristic trichilemmal keratinization.^3,12)^ A dermoid cyst was excluded by the absence of skin appendages, such as hair follicles and sebaceous glands, in the cyst wall.^12)^ A duplication cyst was also excluded because gastrointestinal-type epithelium and organized smooth muscle layers were absent.^12)^ The characteristic trichilemmal keratinization, together with prominent proliferation of squamous epithelium arising from the cyst wall, supported the diagnosis of PTT.^3)^

For cystic lesions extending from the perineum into the retroperitoneal pelvic space, surgical approaches include an abdominal approach, a transperineal approach, or a combined abdominoperineal approach, depending on the anatomical extent of the lesion. In the present case, the marked perineal extension of the giant cyst provided a direct surgical corridor to the retroperitoneal component, enabling tumor resection through the transperineal approach without the need for laparoscopic dissection from the abdominal side. Laparoscopy was subsequently performed for intra-abdominal and pelvic inspection. Furthermore, after releasing the pressure exerted by the overlying perineal skin, the cystic fluid gradually shifted toward the perineal component. This allowed the retroperitoneal component of the cyst wall to progressively collapse and descend into the perineal operative field, facilitating safe circumferential dissection under direct vision. The cystic nature of the lesion was an important factor that enabled transperineal resection in the present case. Although it may be difficult to predict preoperatively whether a giant cystic lesion with deep pelvic extension can be resected entirely from the perineal side, several favorable features may facilitate this approach, including a soft and pliable cyst wall, absence of invasive growth into the surrounding tissues, and relatively small papillary components arising from the cyst wall. These features may allow the pelvic component to collapse and descend into the perineal operative field. In contrast, lesions with a rigid wall, invasive growth into surrounding structures, or large solid components may not collapse or descend caudally in the same manner and may require a combined abdominoperineal approach.

A key technical advantage of this maneuver was the improvement in exposure during cranial dissection. With a conventional transperineal approach, visualization and maneuverability become progressively limited as the dissection proceeds deeper into the pelvis. In the present technique, gravitational redistribution of the cystic contents allowed the pelvic component to collapse and descend into the perineal operative field. Wide dissection between the overlying perineal skin and the cyst wall increased the mobility and expandability of the extracorporeal component, thereby facilitating redistribution of the cystic contents toward the perineal side. Consequently, the dissection plane was brought closer to the surgeon rather than requiring progressively deeper dissection into a narrow pelvic space. This enabled more precise and delicate circumferential dissection under direct vision and represents an important technical advantage of this approach.

From an oncological perspective, preservation of cyst integrity and avoidance of tumor violation are essential when using this approach. In the present case, the well-defined capsule and absence of apparent invasion into adjacent organs on preoperative imaging supported the feasibility of transperineal resection. In addition, direct visualization and tactile feedback during the transperineal procedure allowed the dissection plane and resection margin to be adjusted according to the intraoperative findings. This advantage may also be useful when malignant PTT is suspected. However, when the pelvic component cannot be brought down into the perineal operative field, a combined abdominoperineal approach should be considered. Furthermore, if malignant invasion of the rectum or adjacent pelvic structures is suspected and an adequate oncological margin cannot otherwise be secured, more extensive surgery, including abdominoperineal resection, may need to be considered.

Laparoscopic inspection is not necessarily required after every transperineal resection. However, in cases involving a giant cystic lesion that markedly displaces the pelvic and intra-abdominal organs, as in the present case, unexpected injury during transperineal manipulation may result in serious postoperative complications. In addition, laparoscopic inspection may be useful for confirming the absence of additional intra-abdominal or pelvic lesions, including lesions potentially associated with the cystic lesion and residual cystic or daughter lesions. Therefore, we consider laparoscopic inspection to be a useful adjunct in selected cases.

## CONCLUSIONS

We report an exceptionally rare giant PTT arising in the perineum with retroperitoneal extension. Tumor resection was achieved through the transperineal approach by taking advantage of the marked perineal expansion of the cyst. This technique may represent a useful surgical option for selected noninvasive cystic lesions extending from the perineum into the retroperitoneum.

## DECLARATIONS

### Availability of data and materials

The datasets supporting the conclusions of this article are included within the article.
